# Enhancing SOAP Documentation in Internal Medicine: A Three-Cycle Clinical Audit at Soba University Hospital

**DOI:** 10.7759/cureus.97513

**Published:** 2025-11-22

**Authors:** Maisaa Mohamed, Mohja Yosif, Latifa Mohamed, Tagwa Yassin, Hassan I Osman, Esraa Alem, Hafsa Almogadam, Anas Mohammed Ali Mohammed, Razan Abdalrahim Rabih Fadlelseid, Lamis Elfateh Mohamed Ali, Nuha Mohamed

**Affiliations:** 1 Internal Medicine, Soba University Hospital, Khartoum, SDN; 2 Internal Medicine, Soba University Hospita, Khartoum, SDN; 3 Research, Napata Research and Innovation Center (NRIC), Khartoum, SDN; 4 Internal Medicine, Umluj General Hospital, Umluj, SAU; 5 Obstetrics and Gynaecology, Rabak Hospital, Rabak, SDN; 6 Internal Medicine, Czech Rehabilitation Hospital, Al Ain, ARE

**Keywords:** clinical audit, documentation, follow-up checklist, internal medicine, soap note

## Abstract

Background: High-quality clinical documentation, particularly using the SOAP (Subjective, Objective, Assessment, Plan) format, is essential for patient safety and effective healthcare delivery. In Sudan, inadequate documentation practices have been linked to poor continuity of care and increased risk of medical errors. This study aimed to evaluate and improve the quality of SOAP documentation in the Internal Medicine department at Soba University Hospital, Khartoum, through a structured clinical audit.

Methods: A retrospective, three-cycle clinical audit was conducted using a locally adapted SOAP checklist covering 21 criteria across administrative, subjective, objective, assessment, and plan components. Data were collected from 111 patient records (37 per cycle) between March 2021 and March 2022. Following an initial audit identifying key deficiencies, targeted interventions-including educational seminars, consultant mentorship, standardized templates, and improved equipment provision-were implemented. Compliance rates were compared across audit cycles.

Results: The initial audit revealed significant deficiencies, with only two criteria meeting the ≥80% compliance standard. Post intervention, notable improvements were observed in several areas: social history documentation increased by 48.4%, history of presenting illness by 25.7%, and medication planning reached the 80% standard. However, persistent gaps remained in administrative data, objective components, and allergy documentation. Differential diagnosis documentation showed variability across cycles.

Conclusion: Targeted interventions led to measurable improvements in SOAP documentation quality, particularly in subjective and plan components. Nevertheless, administrative and objective documentation standards remain unmet, highlighting the need for sustained education, standardized processes, and systemic reforms. These findings provide a foundation for broader quality improvement initiatives in Sudanese healthcare settings.

## Introduction

High-quality clinical documentation, especially using the SOAP (Subjective, Objective, Assessment, Plan) format, is fundamental to patient safety, continuity of care, and effective communication among healthcare providers. High-quality clinical documentation is defined as documentation that is complete, accurate, clear, timely, standardized, clinically relevant, and auditable, ensuring effective communication, continuity of care, and patient safety. In Sudan, the quality of medical documentation remains a significant challenge, with studies highlighting issues such as incomplete records, lack of standardization, and insufficient training for healthcare staff [1,2]. All SOAP documentation reviewed in this audit was paper-based, as the hospital did not have an electronic medical record system during the study period.

At Soba University Hospital, Khartoum, and other major Sudanese institutions, the structure and completeness of discharge summaries and clinical notes are often inadequate. This has been linked to poorly designed forms, limited use of standardized templates, and a lack of clear policies and regular staff training [2,3]. National strategies, such as the Sudan National Health Care Quality Policy and Strategy, emphasize the need for comprehensive, high-quality health information and capacity building in documentation practices [3]. 

Clinical audits and research from Sudanese hospitals demonstrate that targeted interventions can improve documentation quality. For instance, a study at Dongola Specialized Hospital found that structured documentation and staff training significantly enhanced the quality of follow-up checklists [4]. Similarly, a two-cycle audit at Rabak Teaching Hospital showed that introducing standardized checklists and educational sessions improved the completeness and accuracy of clinical records [5]. A prospective audit in the pediatric surgery department at National Ribat University Hospital also reported notable improvements after implementing structured templates and training [6]. 

Nursing documentation in Khartoum hospitals reflects similar challenges, with research indicating that while most nurses have good theoretical knowledge, the practical quality of their documentation is poor compared to international standards, mainly due to lack of training, resources, and formal guidelines [1]. Broader reviews of the Sudanese health system further highlight the dynamic interplay between healthcare quality, system organization, and workforce capacity [7]. 

Additionally, the lack of robust health information systems and quality assurance mechanisms contributes to documentation errors and medical mistakes, underlining the urgent need for systemic improvements [8]. Experiences from neighboring countries and fragile health systems, such as South Sudan, reinforce the importance of quality assurance and structured monitoring to improve care delivery [9]. 

This study aimed to answer the research question: How can the quality of SOAP documentation at Soba University Hospital be improved through the use of standardized templates, ongoing staff education, and regular clinical audits [10]? 

By assessing the quality of documentation, we are hoping to identify gaps and deficiencies in documentation and explore practical means to improve documentation. This research could serve as a starting point for future researchers to further explore this subject and extend it to include other hospitals.

## Materials and methods

Study design 

This study employed a retrospective clinical audit design to assess the quality of medical record documentation using the SOAP checklist in the Internal Medicine department at Soba University Hospital, Khartoum. The audit was conducted over three cycles, with data collected from archived medical records. A cross-sectional approach was used to evaluate documentation practices at specific time points, allowing for the identification of gaps and trends in adherence to SOAP standards [11]. The design facilitated a systematic review of documentation quality and provided a basis for targeted interventions [12]. 

The SOAP checklist used was based on open-access standards, adapted from the National Center for Biotechnology Information (NCBI) clinical documentation guidelines and the World Health Organization discharge summary recommendations. Since these resources are publicly available, no formal permission was required, but full attribution has been provided. The checklist used in this audit is provided in the Appendix.

Data collection 

The study was conducted in the internal medicine ward of Soba University Hospital. Data were collected using a locally adjusted SOAP checklist, which included 21 criteria across five sections: administrative data, Subjective, Objective, Assessment, and Plan. Each item was scored as either documented or not documented, and compliance was expressed as a percentage of total records meeting the criterion. The inclusion criteria comprised all medical records from the internal medicine department that fulfilled the SOAP sheet requirements during the specified time periods. Records not affiliated with the internal medicine ward or lacking SOAP documentation were excluded. 

Sampling and phases 

Study Phases and Interventions

First Cycle (Baseline and Root Cause Analysis) - March 21 to April 25, 2021: The baseline assessment revealed substantial deficiencies in documentation. Only two criteria (date of admission and unit documentation) met the ≥80% compliance standard. Major gaps included poor documentation of time of admission, allergy history, and differential diagnosis. Although documentation of differential diagnosis is not mandatory for all clinical cases, it was included in this audit to assess the completeness of clinical reasoning and problem assessment among physicians. A root cause analysis was conducted using focus group discussions and the “5 Whys” approach. This was complemented by a fishbone (Ishikawa) diagram, which categorized contributing factors under People, Process, Equipment, and Policy/Environment. Key issues identified included the absence of standardized templates, insufficient staff training, high turnover of junior doctors, and limited availability of basic medical equipment.

Interventions - May to June 2021: Targeted interventions were developed and implemented to address the identified deficiencies. Educational seminars were delivered to improve awareness of SOAP standards, with emphasis on the clinical and medico-legal importance of complete documentation. Senior consultants were actively involved in mentoring junior staff. A standardized SOAP checklist was introduced to promote consistency in documentation. In addition, the hospital management facilitated the provision of essential equipment, such as pulse oximeters, to support accurate recording of vital signs.

Second and Third Cycles (Post-Intervention Evaluation) - May to June 2021 and February to March 2022: The impact of the interventions was evaluated during the second and third cycles. Improvements were observed in several areas, particularly in history of presenting illness, social history, and medication planning, all of which demonstrated significant progress toward the compliance standard. However, persistent deficiencies remained in administrative data, allergy documentation, and certain objective components, reflecting the ongoing challenges posed by staff turnover and workflow limitations.

Data analysis

Data were analyzed using Microsoft Excel. Descriptive statistics (frequencies and percentages) were used to compare compliance rates across cycles. Trends were identified to assess the effectiveness of interventions. 

## Results

Throughout the three audit cycles, a total of 111 BSOAP (Background, Subjective, Objective, Assessment, Plan) follow-up sheets were analyzed, with 37 sheets reviewed in each cycle. These sheets are paper-based ward-round documentation forms structured according to the SOAP format used in daily inpatient follow-up. The compliance rates showed variable improvement across different documentation components when compared to the ≥80% standard. 

The administrative data components demonstrated mixed results (Table 1). While Date of Admission maintained high compliance (94.3% in Cycle 1 to 82.8% in Cycle 3), it showed an 11.5% decline. In contrast, Time of Admission documentation improved significantly by 39.1%, rising from 5.7% to 44.8%. Doctor's Name/Signature and Unit Documentation remained relatively stable but below the standard throughout all cycles. 

**Table 1:** Administrative and subjective components compliance Compliance rates for administrative and subjective SOAP components across three audit cycles (n=37 per cycle). Results are presented as the number of records meeting each criterion, with percentages in parentheses. Compliance was compared against the ≥80% audit standard. Δ represents the net change between Cycle 1 and Cycle 3. SOAP: Subjective, Objective, Assessment, Plan

#	Criteria	Cycle 1 (n=37)	Cycle 2 (n=37)	Cycle 3 (n=37)	Standard	Δ (C1→C3)
Administrative Data						
1	Date of Admission	35 (94.3%)	31 (83.8%)	31 (82.8%)	≥80%	-11.5%
2	Time of Admission	2 (5.7%)	4 (10.8%)	17 (44.8%)	≥80%	+39.1%
3	Doctor’s Name/Signature	20 (54.3%)	22 (59.5%)	19 (51.7%)	≥80%	-2.6%
4	Unit Documentation	31 (82.9%)	28 (75.7%)	31 (82.8%)	≥80%	-0.1%
Subjective Data						
5	Chief Complaint	28 (74.3%)	32 (86.5%)	28 (75.9%)	≥80%	+1.6%
6	History of Presenting Illness	21 (57.1%)	30 (81.1%)	31 (82.8%)	≥80%	+25.7%
7	Medical History	19 (51.4%)	30 (80.6%)	27 (72.4%)	≥80%	+21.0%
8	Surgical History	16 (42.9%)	29 (77.8%)	25 (65.5%)	≥80%	+22.6%
9	Social History	8 (20.6%)	22 (59.5%)	26 (69.0%)	≥80%	+48.4%
10	Review of Systems	14 (37.1%)	15 (40.5%)	26 (69.0%)	≥80%	+31.9%
11	Current Medications	19 (51.4%)	24 (64.9%)	26 (69.0%)	≥80%	+17.6%
12	Known Allergies	3 (8.8%)	19 (51.4%)	17 (44.8%)	≥80%	+36.0%

For subjective components (Table 1), Social History showed the most remarkable improvement (48.4% increase from 20.6% to 69.0%). Other notable improvements included History of Presenting Illness (25.7% increase), Medical History (21.0% increase), and Review of Systems (31.9% increase). However, Known Allergies, despite a 36.0% improvement, remained below the desired standard at 44.8% in Cycle 3. 

The objective components (Table 2) revealed modest gains: Investigations improved by 21.6% (from 54.3% to 75.9%), while Vital Signs and Physical Examination showed minimal changes (10.0% and 1.5% improvements respectively). All objective components failed to meet the 80% standard by Cycle 3. 

**Table 2:** Objective and assessment components compliance Compliance rates for objective and assessment SOAP components across three audit cycles (n=37 per cycle). Each criterion was assessed for documentation completeness. Compliance was measured against the ≥80% standard. Δ indicates the percentage change between Cycle 1 and Cycle 3. SOAP: Subjective, Objective, Assessment, Plan

#	Criteria	Cycle 1 (n=37)	Cycle 2 (n=37)	Cycle 3 (n=37)	Standard	Δ (C1→C3)
Objective Data						
13	Vital Signs	15 (40.0%)	14 (37.8%)	19 (50.0%)	≥80%	+10.0%
14	Physical Examination	22 (57.1%)	23 (62.2%)	22 (58.6%)	≥80%	+1.5%
15	Investigations	20 (54.3%)	22 (59.5%)	29 (75.9%)	≥80%	+21.6%
Assessment Data						
16	Provisional Diagnosis	27 (71.4%)	27 (73.0%)	26 (69.0%)	≥80%	-2.4%
17	Differential Diagnosis	7 (17.1%)	27 (72.2%)	19 (51.0%)	≥80%	+

In the assessment components (Table 2), documentation primarily reflected the provisional or confirmed diagnosis rather than multiple differential diagnoses, showing wide variability (17.1% in Cycle 1, 72.2% in Cycle 2, then 51.0% in Cycle 3). Differential diagnoses were rarely recorded in the reviewed notes.

The plan components (Table 3) displayed the most consistent improvements: Medication Plan compliance increased by 38.1% to reach exactly 80% in Cycle 3, while Follow-Up Instructions improved by 27.0%. However, Consultations showed an 11.5% decline in compliance.

**Table 3:** Plan components compliance Compliance rates for plan components of SOAP documentation across three audit cycles (n=37 per cycle). Values represent the number of records with documented criteria (percentage in parentheses). Compliance threshold was set at ≥80%. Δ indicates change between Cycle 1 and Cycle 3. SOAP: Subjective, Objective, Assessment, Plan

#	Criteria	Cycle 1 (n=37)	Cycle 2 (n=37)	Cycle 3 (n=37)	Standard	Δ (C1→C3)
18	Additional Testing	17 (47.1%)	12 (32.4%)	18 (48.3%)	≥80%	+1.2%
19	Consultations	16 (42.5%)	12 (32.4%)	12 (31.0%)	≥80%	-11.5%
20	Medication Plan	18 (41.9%)	22 (59.4%)	30 (80.0%)	≥80%	+38.1%
21	Follow-Up Instructions	15 (40.0%)	20 (54.0%)	25 (67.0%)	≥80%	

These findings demonstrate that while certain documentation elements (particularly in subjective data and medication planning) responded well to interventions, other critical components (especially in administrative and objective data) require additional focus to meet compliance standards. The variability in Differential Diagnosis documentation suggests particular attention should be paid to maintaining consistent improvements in assessment components.

## Discussion

This audit assessed the quality of SOAP documentation in the Internal Medicine department at Soba University Hospital, Khartoum, revealing both progress and persistent challenges following targeted interventions. The findings are consistent with broader evidence from Sudanese and international literature, which highlight the critical role of documentation in patient safety, care continuity, and interprofessional communication [1,2,11]. 

Key improvements and ongoing challenges 

Interventions such as educational seminars, consultant mentorship, standardized templates, and improved equipment availability led to notable improvements in several documentation components, particularly in subjective data (e.g., history of presenting illness, social history) and medication planning. These results align with similar studies from Sudanese hospitals, such as those conducted at Dongola Specialized Hospital and Rabak Teaching Hospital, which demonstrated that structured documentation and staff training significantly enhance the completeness and accuracy of clinical records [4,9]. A prospective audit in the pediatric surgery department at National Ribat University Hospital also reported notable improvements after implementing structured templates and training [10]. 

However, despite these gains, critical areas such as administrative data, objective components (vital signs, physical examination), and allergy documentation remained below the 80% compliance standard, echoing findings from other local audits and reports on Sudanese healthcare [2,8]. Nursing documentation in Khartoum hospitals reflects similar challenges, with research indicating that while most nurses have good theoretical knowledge, the practical quality of their documentation is poor compared to international standards, mainly due to lack of training, resources, and formal guidelines [2]. 

Systemic and contextual factors 

The root cause analysis identified systemic issues including the lack of standardized templates, insufficient training, high staff turnover, and limited availability of medical equipment. These challenges are not unique to Soba University Hospital; they mirror findings from studies in Khartoum and other Sudanese institutions, as well as broader analyses of the Sudanese health system post-revolution, which emphasize the dynamic interplay between workforce capacity, system organization, and healthcare quality [5,7]. The lack of robust health information systems and quality assurance mechanisms further compounds these issues, increasing the risk of documentation errors and medical mistakes [1,8]. 

International and regional comparisons 

Blair et al. emphasize that documentation reflects professional standards and patient safety, and the literature highlights that continuous education for nursing and medical staff is a crucial driver of sustained improvements in documentation quality [13,14]. International evidence supports the need for ongoing education, standardized documentation, and continuous quality improvement, as studies from Uganda, Ethiopia, and Iran indicate that resource and training limitations are common barriers to high-quality documentation, though targeted interventions can yield significant improvements [15,16].

Policy and practice implications 

The Sudan National Health Care Quality Policy and Strategy, alongside international recommendations, advocate for comprehensive health information systems, capacity building, and regular clinical audits [3]. The results of this audit, as well as those from similar studies in Sudan and the region, underscore the importance of integrating these strategies into routine practice [4,9,10,13-17]. Addressing the identified gaps will require not only ongoing staff training and the use of standardized templates but also investment in infrastructure and the establishment of regular audit cycles. 

Limitations 

This study is limited by its single-center, retrospective design, which may affect the generalizability of the findings. Future research should expand to include multiple departments and hospitals to provide a broader perspective on documentation quality in Sudan [1,2,5]. 

## Conclusions

While targeted interventions have improved the quality of SOAP documentation at Soba University Hospital, significant challenges remain, particularly in administrative and objective data. These findings are consistent with both Sudanese and international literature, which collectively highlight the need for sustained efforts, ongoing education, and systemic improvements to achieve and maintain high-quality clinical documentation. This study provides a foundation for future research and quality improvement initiatives in Sudanese healthcare settings.

## Appendices

**Table 4:** SOAP documentation checklist SOAP: Subjective, Objective, Assessment, Plan

Section	Criteria	Yes (✓)	No (X)	N/A
A. Administrative Data	1. Date of Admission			
	2. Time of Admission			
	3. Doctor’s Name / Signature			
	4. Unit Documentation			
B. Subjective Data	5. Chief Complaint			
	6. History of Presenting Illness			
	7. Medical History			
	8. Surgical History			
	9. Social History			
	10. Review of Systems			
	11. Current Medications			
	12. Known Allergies			
C. Objective Data	13. Vital Signs			
	14. Physical Examination			
	15. Investigations			
D. Assessment Data	16. Provisional Diagnosis			
	17. Differential Diagnosis			
E. Plan Data	18. Additional Testing			
	19. Consultations			
	20. Medication Plan			
	21. Follow-Up Instructions			

## References

[1] Elhadi YA (2022). Medical errors in Sudan: a call for urgent investigation and action. Ann Med Surg (Lond).

[2] Ali AM, Albashir WA, Mariod A (2020). Nursing documentation in selected hospitals in Khartoum State-Sudan. J Int Health Sci Manag.

[3] Federal Ministry of Health, Republic of Sudan (2017). Sudan National Health Care Quality Policy and Strategy 2017. https://platform.who.int/docs/default-source/mca-documents/policy-documents/policy/SDN-CC-31-01-POLICY-2017-eng-National-Health-Care-Quality.pdf.

[4] Muhammed A, Abdalgadir Hamdnaalla MA, Fakher Aldeen Noman FA (2024). Evaluating and improving the quality of the follow-up checklist at Dongola specialised hospital: a clinical audit. Cureus.

[5] Hassanain SA, Eltahir A, Elbadawi LI (2022). Freedom, peace, and justice: a new paradigm for the Sudanese health system after Sudan's 2019 uprising. ERF.

[6] Berendes S, Lako RL, Whitson D, Gould S, Valadez JJ (2014). Assessing the quality of care in a new nation: South Sudan's first national health facility assessment. Trop Med Int Health.

[7] KIT Institute (2020). Access to Health Care in South Sudan: A Qualitative Analysis Report. https://www.kit.nl/wp-content/uploads/2021/09/HPF3-Access-to-healthcare-study-A-qualitative-analysis-report.pdf.

[8] World Bank (2012). Development of a medical waste management Plan for South Sudan. https://documents1.worldbank.org/curated/en/473271468000911143/pdf/E41280EA0P132101300SS0MW0Final0Rep.pdf.

[9] Muhammed A, Modawy Alkheder MA, Hamadelniel Alhadi IA (2025). Improving the quality of follow-up checklists in Rabak Teaching Hospital: a two-cycle clinical audit. Cureus.

[10] Ibrahim MY, Koko A, Abdelraouf Ahmed A (2025). Improving pediatric surgical follow-up documentation: a prospective clinical audit in the department of pediatric surgery at National Ribat University Hospital, Sudan. Cureus.

[11] Donabedian A (1988). The quality of care. How can it be assessed?. JAMA.

[12] Cameron DB, Rangel SJ (2016). Quality improvement in pediatric surgery. Curr Opin Pediatr.

[13] Blair W, Smith B (2012). Nursing documentation: frameworks and barriers. Contemp Nurse.

[14] Alkouri Alkouri, OA OA, AlKhatib AJ, Kawafhah M (2016). Importance and implementation of nursing documentation: review study. ESJ.

[15] Vafaei SM, Manzari ZS, Heydari A, Froutan R, Farahani LA (2018). Improving nursing care documentation in emergency department: a participatory action research study in Iran. Open Access Maced J Med Sci.

[16] Nakate G, Dahl D, Petrucka P, Drake KB, Dunlap R (2015). The nursing documentation dilemma in Uganda: neglected but necessary. A case study at Mulago National Referral Hospital. Open Journal of Nursing.

[17] Molla F, Temesgen WA, Kerie S, Endeshaw D (2024). Nurses’ documentation practice and associated factors in eight public hospitals, Amhara Region, Ethiopia: a cross-sectional study. SAGE Open Nurs.

